# Supplementary material for: Dysregulation of ErbB4 Signaling Pathway in the Dorsal Hippocampus after Neonatal Hypoxia-Ischemia and Late Deficits in PV+ Interneurons, Synaptic Plasticity and Working Memory
Source: Int J Mol Sci. 2022 Dec 28;24(1):508. doi: 10.3390/ijms24010508 (PMC9820818; doi:10.3390/ijms24010508)
Supplement: Supplementary file 1 [file ijms-24-00508-s001.zip › Supplementary Table S1.pdf]

SUPPLEMENTAL Table S1

List of primary antibodies for immunofluorescence immunohistochemistry & western blotting

Target	Catalog #	Company	Clonality Isotype	Host	MW (kDa) on WB	IHC*	WB
**Parvalbumin**	**NBP2-50036**	Novus	Polyclonal IgY	Chicken	NS	Yes (IF)	No
	**NB120-11427**	Novus	Polyclonal IgG	Rabbit	12	Yes (DAB)	No
**Nrg-1**	**66492-1-Ig**	ProteinTech	Monoclonal IgG1	Mouse	67	Yes (IF)	No
**ErbB4**	**4795S**	Cell Signaling	Monoclonal IgG	Rabbit	150	No	Yes
	**GTX80811**	GeneTex	Monoclonal IgG2b	Mouse	150	Yes (IF)	
**Phospho (Tyr 1162) ErbB4**	**PA5-12606**	ThermoFisher	Polyclonal IgG	Rabbit	150	No	Yes
**Akt**	**2920S**	Cell Signaling	Monoclonal IgG1	Mouse	60	No	Yes
**Phospho (Ser 473) Akt**	**9271S**	Cell Signaling	Polyclonal IgG	Rabbit	60	No	Yes
**Syt2**	**808402**	DSHB	Monoclonal IgG2a	Mouse	60	Yes (IF)	No
**Vglut2**	**ab216463**	Abcam	Monoclonal IgG	Rabbit	64	Yes (IF)	No
**GFAP**	**16825-1-AP**	ProteinTech	Polyclonal IgG	Rabbit	50	No	Yes
	**Z0334**	DAKO North America	Polyclonal IgG	Rabbit	NS	Yes (DAB)	No
**α-fodrin**	**FG6090**	Enzo Life Sciences	Monoclonal IgG1	Mouse	250, 150, 120	No	Yes

ErbB4, erb-b2 receptor tyrosine kinase 4; **GFAP,** Glial fibrillary acidic protein; **Nrg1,** Neuregulin 1; **Phospho**, phosphorylated form; **WB,** Western Blot; **NS,** Not suitable for western blots; **MW,** Molecular Weight. *, post WB validation.

Negative controls for immunofluorescence. The used subtypes and species-specific immunoglobin were as follows:

i. Rabbit IgG isotype (Vector Laboratories, Inc; Burlingame, CA, USA; RRID: AB_2336355)
ii. Mouse IgG isotype (Vector Laboratories, Inc; RRID: AB_2532954)
iii. Chicken IgY isotype (R&D Systems; RRID: AB_354263)

Additional blocking for IHC-IF. Goat F(ab) Anti-Mouse IgG H&L. (Jackson ImmunoResearch 115-007-003; RRID: AB_2338476) Goat secondary antibody prepared via immunoaffinity chromatography using Mouse IgG coupled agarose beads against a monospecific antiserum. Additional blocking when performing IF protocols that involved Mouse IgG antibodies was used on mice that exhibited heavy injury.
