# Supplementary figures and images for: Dysregulation of ErbB4 Signaling Pathway in the Dorsal Hippocampus after Neonatal Hypoxia-Ischemia and Late Deficits in PV+ Interneurons, Synaptic Plasticity and Working Memory
Source: Int J Mol Sci. 2022 Dec 28;24(1):508. doi: 10.3390/ijms24010508 (PMC9820818; doi:10.3390/ijms24010508)

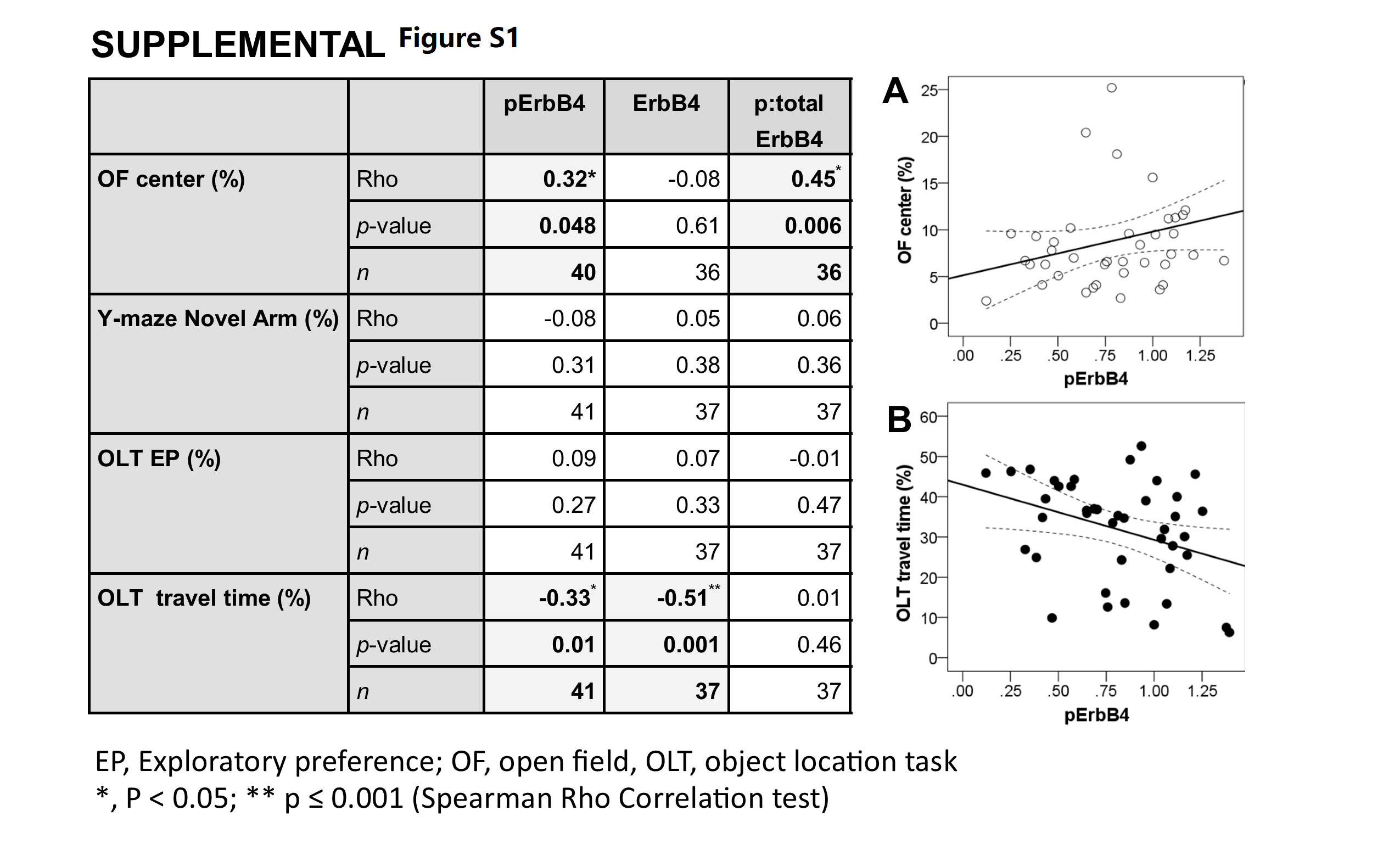

Supplement: Supplementary file 1 [file ijms-24-00508-s001.zip › Supplementary Figure S1.tif]
